# Edinburgh Royal Infirmary

**Published:** 1830-10

**Authors:** 


### EDINBURGH ROYAL INFIRMARY.

### *Aneurism by Anastomosis on the Face, cured by Ligature.*

Robert Milligan, æt. nineteen, admitted under the care of Mr. Liston, May 11th.

A livid projecting tumor, presenting all the usual characters of aneurism by anastomosis, occupies the middle of the upper lip, almost the whole of the nose, and the lower part of the forehead, producing little inconvenience to the patient, but causing unseemly disfiguration of his countenance. The frontal, nasal, and labial portions of the tumor, appear to be circumscribed and separate; but, when examined, are found to be intimately connected with each other.

The patient states, that a small blue speck existed at birth on the left side of his nose, and that this nævus increased gradually, and without causing any pain, until it attained its present size and situation: of late it has been almost stationary. Several livid spots are seen on other parts of his body: one on the chin, another on the breast, and on the inside of the left foot: these, however, are extremely minute, and are not enlarging.

May 12th.—Mr. Liston included the labial portion of the tumor in two strong ligatures, which were passed beneath the diseased part by means of curved needles in fixed handles.

By the 18th the enclosed mass had become completely sphacelated, and was removed.

On the 19th the frontal portion had its vascular supply interrupted by means of ligatures, applied in the same way as formerly, and the diseased part soon separated.

The remaining and largest part of the tumor, that involving the nose, was also surrounded by ligatures; but, from the largeness of its size, and the peculiar situation which it occupied, the operation was necessarily more tedious than in the preceding instances. By means of fixed needles, insinuated carefully beneath the swelling, with their concave surfaces towards the prominence of the nose, numerous ligatures were passed, and were so disposed that, after tying the extremity of each with the one next it, the tying of the last tightened the whole. The point of the nose was, by their application, drawn slightly upwards; but on the separation of the sloughs it regained its usual situation. The tumor was completely surrounded, and soon showed symptoms of rapid decay.

Mr. Liston remarked, that from the great size of the growth, and the apparent activity of the congeries of vessels which composed it, he believed an attempt to remove it by incision would have been extremely hazardous; but that the employment of the ligature was a safe and effectual practice.

An attack of erysipelas followed the application of the ligatures to each of the swellings, and involved the greater part of the face, commencing in the neighbourhood of the tumor, and rapidly extending around. It soon yielded, however, to the employment of punctures and warm fomentations, and the internal exhibition of antimonial medicines. The predisposing cause of erysipelas seemed to be the particular condition of the atmosphere at that season, for it was prevalent both in the rest of the house and in private practice.

After the separation of the sloughs, the surface assumed a healthy appearance, forming small florid granulations, and discharging a moderate quantity of good pus. When the patient left the hospital, the wound had contracted to a small size, and cicatrization was proceeding rapidly. His appearance was much improved.

